# Evidence does not confirm that lithium prevents suicide: a reply to Bschor *et al*.

**DOI:** 10.1017/S2045796022000737

**Published:** 2022-12-13

**Authors:** Joanna Moncrieff, Martin Plöderl, Zainab Nabi, Jacki Stansfeld, Lisa Wood

**Affiliations:** 1 Division of Psychiatry, University College London, London, UK; 2 Christian-Doppler-Klinik, Salzburg, Austria; 3 Paracelsus Medizinische Privatuniversitat, Salzburg, Austria

Bschor *et al*.'s conclusion that the anti-suicidal effect of lithium is ‘well-established’ is not justified, and is challenged even by other proponents of lithium (Baldessarini and Tondo, 2022). We conducted an updated meta-analysis of data on suicide from randomised trials of lithium because previous meta-analyses have excluded the majority of recent data due to using the Peto method, which cannot handle trials with zero events Therefore, these meta-analyses were based on small numbers of participants – 244 on lithium and 241 on placebo in the meta-analysis of placebo-controlled trials by Cipriani *et al*. (Cipriani *et al*., 2013), for example. In the case of an infrequent outcome like suicide, it is now recognised that including data from trials with zero events leads to more reliable estimates (Ren *et al*., 2019). Our analyses were based on data from 1278 people allocated to lithium and 1300 on placebo.

Concluding that lithium has anti-suicidal effects involves relying on older and thus potentially unreliable studies or lower-level evidence from observational studies and, ignoring studies without events, potential biases in the literature that usually lead to overestimations of treatment effects and results for suicide attempts. But even with such an approach, uncertainty remains high due to the low overall number of suicides and wide confidence intervals.

We excluded trials published prior to 2000 in our main analysis because there is evidence that suicide was not reliably reported in these trials. In the trial by Glen *et al*. (Glen *et al*., 1984), for example, no suicides were reported, but one was later revealed. We believe an analysis that includes all the data from higher quality trials published from 2000 is more reliable than one that includes a small number of trials of variable quality mostly published in the 1960s and 1970s. However, cognisant that this would be a likely criticism, we conducted a sensitivity analysis including trials published prior to 2000 which produced similar results to the main analysis.

We believe it is a reasonable assumption that suicides would be reported if any occurred in trials published from 2000. However, there was only one trial published in this period that did not specifically report on suicides, or for which data was unobtainable, and we performed a sensitivity analysis excluding this trial which also produced a similar result to the main analysis.

We did not include trials that compared lithium with an active comparator because whether lithium performs better or worse than an active comparator is a different question from whether it outperforms placebo or treatment as usual. If an effect exists, it needs to be demonstrated against placebo or usual treatment. Indeed, although Cipriani *et al*. (2013) searched for active comparator trials, they performed separate meta-analyses for trials that compared lithium with placebo and those that used active comparators.

In line with this, we did not include the trials by Greil *et al*., because they employed an active comparator, comparing lithium with carbamazepine, and did not have a placebo or treatment as usual group.

In the study by Katz *et al*. (Katz *et al*., 2022), three deaths were reported in the placebo group, as noted by Bschor *et al*., but only one was a suicide that occurred during the trial. One was an opioid overdose that was not classified as a suicide by the authors, and one was a suicide that occurred one month after the end of the trial (and was only detected much later). In order to be consistent with other studies (which may have found suicides that occurred after the trial and not reported them), we did not include this event and the authors did not include it in their analysis either.

Bschor *et al*., recommend using naturalistic data to evaluate the effect of lithium on suicide but this is problematic for various reasons. First, people who adhere to any treatment are generally healthier and have better outcomes than those who do not (Curtis *et al*., 2011), and a treatment like lithium that requires regular blood tests is likely to reinforce this ‘healthy complier’ effect. Second, people with a high risk of suicide are less likely to be treated with lithium due to its high toxicity. Finally, suicide risk is elevated in the year following lithium withdrawal (Baldessarini *et al*., 1999), which is likely to be partially due to a withdrawal-specific effect, which may inflate suicide rates in people classified as ‘untreated’. Although we did not find evidence of increased suicides in trials that involved lithium withdrawal, these trials had excluded those at high risk of suicide.

Bschor *et al*., also cite data presented by Baldessarini and Tondo's (2022), but this was not a systematic review or meta-analysis, and involved selected data combining suicides and suicide attempts, but excluded suicide attempts in some of the trials presented for no clear reason.

Finally, we draw attention again to the fact that our results, which are based on a relatively large sample derived from modern randomised trials, are consistent with those from the large, high-quality, randomised trial designed to evaluate the efficacy of lithium in preventing suicidal behaviour, which was stopped early due to lack of effect (Katz *et al*., 2022). Baldessarini and Tondo (2022) also recently acknowledged the uncertainty of lithium's anti-suicidal properties, describing how : ‘recruiting participants to such trials [suicide prevention trials of lithium] may be made difficult by an evidently prevalent belief that the question of antisuicidal effects of lithium is already settled, which it certainly is not.’ (Baldessarini and Tondo, 2022) (p. 10).
